# Criteria for Selecting Suitable Infectious Diseases for Phage Therapy

**DOI:** 10.3390/v10040177

**Published:** 2018-04-05

**Authors:** David R. Harper

**Affiliations:** Evolution Biotechnologies, Colworth Science Park, Sharnbrook, Bedfordshire MK44 1LZ, UK; drh@evolutionbiotech.com; Tel.: +44-1234-818312

**Keywords:** bacteriophage, therapy, phage therapy, bacterial disease, infection, target selection

## Abstract

One of the main issues with phage therapy from its earliest days has been the selection of appropriate disease targets. In early work, when the nature of bacteriophages was unknown, many inappropriate targets were selected, including some now known to have no bacterial involvement whatsoever. More recently, with greatly increased understanding of the highly specific nature of bacteriophages and of their mechanisms of action, it has been possible to select indications with an increased chance of a successful therapeutic outcome. The factors to be considered include the characteristics of the infection to be treated, the characteristics of the bacteria involved, and the characteristics of the bacteriophages themselves. At a later stage all of this information then informs trial design and regulatory considerations. Where the work is undertaken towards the development of a commercial product it is also necessary to consider the planned market, protection of intellectual property, and the sourcing of funding to support the work. It is clear that bacteriophages are not a “magic bullet”. However, with careful and appropriate selection of a limited set of initial targets, it should be possible to obtain proof of concept for the many elements required for the success of phage therapy. In time, success with these initial targets could then support more widespread use.

## 1. Introduction

**Figure 1:**
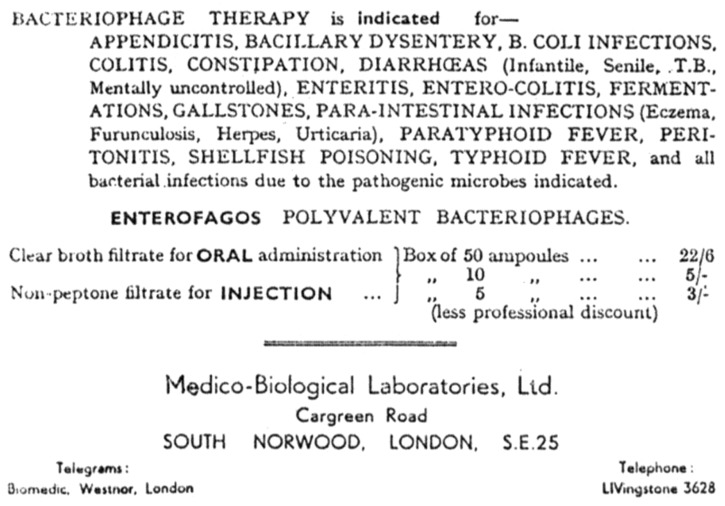
An advertisement for therapeutic bacteriophages from the 1920s (reprinted with the kind permission of Dr. J. Soothill).

When bacteriophages were first used as therapeutic agents from 1919 onwards [1] this was done in the context of an extremely limited knowledge of bacteriophage biology. Given the toxicity of antibacterial agents at the time, which included both mercury and arsenic, there was a clear need for new approaches. Unfortunately, many early uses of phage therapy were driven more by commercial pressures than by science. As a result, bacteriophages were used for a wide variety of indications, many of which had no bacterial component (Figure 1).

Unsurprisingly, with such indications as urticaria and herpes, there was unlikely to be therapeutic benefit due to the effects of bacteriophages. In addition, early bacteriophage preparations contained a large amount of bacterial debris which had a range of immunomodulatory effects, which were considered likely to be responsible for many of the observed clinical effects [2,3]. When data from 100 papers published in the early years of phage therapy was analysed for the American Medical Association, it was concluded that only a very few indications showed good evidence of beneficial effects, with convincing data only for *Staphylococcal* skin disease and some instances of cystitis [2]. With a very limited understanding of the nature of the agent, controversy over its effects, and complications arising from the crude nature of the early therapeutic preparations, it is perhaps unsurprising that phage therapy fell into disuse once chemical antibiotics became widely available. However, as antibiotic resistance changed from an abstract concern to a full-blown crisis [4], interest in phage therapy has been revived [5,6]. This drew on the much greater understanding of bacteriophages resulting from their use during that intervening period in experimental studies, notably in the area of molecular biology.

With this increased understanding of bacteriophage biology came the opportunity for far more precision in their use [5]. Key properties of bacteriophages that had been clarified by this point included many aspects of their biology, such as their nature as bacterial viruses, their highly specific host requirements, and the molecular processes of infection. Technical advances also allowed the purification of bacteriophages away from bacterial contaminants, including the removal of highly active host cell components such as endotoxins. While we are still a long way from a full understanding of the biology of these complex viruses, we can now build on what we do know to use these unique agents far more effectively.

## 2. Selection of Therapeutic Approaches

### 2.1. Clinical Need

When a serious disease cannot be controlled by current therapies there is a need for new approaches. Much of the current interest in phage therapy is driven by concerns over antimicrobial drug resistance, and a working group evaluating a broad range of alternative approaches has concluded that phage therapy is a promising way to counter this issue [7]. There is also some evidence of both natural and engineered bacteriophages driving bacteria towards the loss of existing antibiotic resistance [8,9,10], suggesting a possible role for combinations of bacteriophages with conventional antibiotics.

The initial motivation for the selection of a therapeutic target is typically that there is an unmet need for control of a bacterial infection—that is, a lack of effective antibacterial approaches for a disease with serious medical or economic consequences. If effective controls exist there is rarely a driver for the development of novel therapies, except perhaps to provide an alternative due to commercial drivers. A therapy can be targeted at infections of humans, animals, or plants; conditions addressed by the use of bacteriophages range from ear infections in humans [11] to rot in harvested potatoes [12]. However, the focus of this article is on therapeutic uses and, thus, on unmet clinical need in humans (and, to an extent, animals). Lack of an effective therapy can arise from multiple factors, including evolved antimicrobial resistance or inherent resistance determinants, such as growth in biofilms [13,14]. Thus, unmet need underpins the development of new therapies and generates funding (whether as grants or commercial investment) that is necessary to undertake such work.

No phage therapy has yet been approved for market by the European Medicines Agency (EMA) in the EU or the Food and Drugs Administration (FDA) in the USA. Until this is achieved, phage therapy remains an experimental approach in these jurisdictions, however accepted it is in some other areas such as Eastern Europe. Thus, it is of critical importance to maximise the risk of success in development efforts, since failures can delay, or even destroy, the prospects for developing such approaches. An example of this is gene therapy, which is only now recovering from some early high-profile setbacks [15].

### 2.2. Key Elements of the Disease Target

**Sufficient unmet need:** While individual patients might be in dire need of a particular therapy, there generally need to be enough potential users to justify the costs of development and commercialisation. As an example, many early gene therapy treatments were directed at indications where there were only very small numbers of affected individuals. These “ultra-orphan” targets can help with approval, but the first gene therapy to be approved in Europe, Glybera, targeted a disease which occurs in less than one in a million people and had a consequently high price. The drug was withdrawn five years after launch, having been used just once [16].

**Antimicrobial resistance:** The driver for much of the interest in phage therapy is antimicrobial resistance, where infections may be largely or even completely resistant to conventional antibiotics. Bacteriophages, with their entirely different modes of action, are unaffected by such resistance. Bacteriophages can and will evolve to counter the evolution of bacteriophage resistance by the target bacteria [17]. Another major cause of resistance to conventional antibiotics is bacterial growth in biofilms, which can decrease bacterial sensitivity to antibiotics by over a thousand-fold [13,14]. Bacteriophages have unique capabilities in attacking bacteria in such a setting by targeting both the biofilm matrix and specialized cells growing within it [14]. Efficacy against biofilms has been demonstrated both in vitro and in vivo [11,14] and has the potential to be a significant driver for the adoption of phage therapy.

**Disease results from bacterial infection:** It (nowadays) goes without saying that the target for phage therapy should be bacterial in nature. Bacteriophages are highly specific, with only a relatively small number even crossing species boundaries. They are almost completely inert towards other cells. The days when herpes or urticaria could be regarded as a viable target are, fortunately, long gone.

**Infection is caused by one type of bacteria or a small number of types:** Bacteriophages are often referred to as “exquisitely specific” since most are able to infect and replicate in only a subset of strains within a single bacterial species, meaning that a mixture of bacteriophages (usually referred to as a “cocktail”) is usually needed to target even a single bacterial species, though there are exceptions [5,18]. Thus, a polymicrobial infection is poorly suited to phage therapy. This is a very important difference to the historical use of broad spectrum chemical antibiotics. Although some broad spectrum bacteriophage treatments have been used in Eastern Europe, these contain extremely high numbers of relatively undefined bacteriophages, which would make approval by EMA or FDA complex. An early polymicrobial infection may change during treatment with conventional antibiotics, with a much more limited range of bacteria in the later stages of infection [11]. Such infections offer both unmet need (having failed to resolve with conventional treatments) and a limited range of bacterial targets. They are, thus, well suited to phage therapy approaches.

**Bacteria causing the infection are identified:** Unlike chemical antibiotics, it is unlikely that there will be broad spectrum phage therapeutic cocktails without an extremely complex regulatory process and prohibitive production costs. This is likely to limit first-line use, unless such a treatment is paired with a rapid point-of-care diagnostic test. A simpler approach, at least initially, is to focus on cases where the bacterial target has been identified but is resistant to clearance by existing methods. This was the approach taken in the only phase 2 trial to date to report positive results [11] by targeting late stage ear infections where one bacterial species (*Pseudomonas aeruginosa*) predominates. Additionally, testing must be able to identify and quantify the target bacteria in the intended recipients, both prior to and as part of the trial process.

**Bacteria targeted are responsible for the clinical pathology**: Given the specificity of bacteriophages, simply removing one component of a polymicrobial infection may not result in a positive clinical outcome. While regulators may accept microbiological endpoints (reduction of target bacterial numbers) in initial trials, in later work, improvement in clinical symptoms is likely to be required for trial success. This has been an issue with some work where positive outcomes were not observed [19].

**Potential for useful preclinical work:** While in vitro data is useful, good preclinical data derived from in vivo work is extremely useful in making the case for progression into human trials. This can either be from model systems [20,21] or from analogous infections in animals [11,22,23]. Where no suitable system is available, data collection to permit trials is likely to be more complex.

**Suitability for clinical trials:** Patients must be both available and accessible, in line with current ethical practices. If the patient group requires simultaneous treatment with other antibacterial agents (as is often the case with seriously ill patients, for example, under “expanded access” single patient uses) this can greatly complicate the interpretation of results, reducing the value of the data generated. There are, of course, also many issues relating to the design and conduct of clinical trials, from liaison with regulators to selection of endpoints. However, these fall outside the scope of this article. Nevertheless, it should be noted that even the most promising approach will not succeed with a poorly designed trial.

**Site of infection is accessible:** Bacteriophages are large nucleoprotein structures in the megadalton to gigadalton range. Chemical antibiotics are far smaller. This can lead to misunderstandings over how best to administer bacteriophages among those more used to existing approaches. Given the simplicity of delivery, attention has been focused on topical administration where it is possible to deliver bacteriophages directly to the infected site [11,22,23,24,25,26]. Unlike conventional antibiotics, bacteriophages are unlikely to be able to cross many barriers within the body with useful levels of efficiency, at least when these barriers are intact. These can be normal elements of the body, such as the gut wall or the blood-brain barrier, or part of the pathology of the infection, such as lung tubercles in tuberculosis [27] or closed comedones in acne [28]. However, the ability of bacteriophages to amplify exponentially from very small initial doses [23] can allow even limited bacteriophage numbers to produce a strong localized therapeutic effect.

**Bacterial numbers are sufficient to support amplification:** Localised amplification where their target is present is a unique feature of bacteriophages and underlies their proposed use as therapeutics in most cases. However, in order to support such amplification, there needs to be a sufficient supply of susceptible bacteria [29]. While this is more complex in vivo, it is clear that in some applications, particularly those with cleaned or disinfected sites of infection, bacterial densities may be insufficient to rely on in situ phage amplification [26]. Any therapeutic effect would thus be reduced. In contrast, even very low levels of bacteriophage can produce rapid and dramatic effects when bacterial numbers are high [23]. Thus, phage therapy is better suited to high density bacterial infections, which is, again, counterintuitive for those used to conventional antibiotics.

**Limitations of oral dosing:** Oral dosing is considered highly desirable for conventional antibiotics but is inherently limited for bacteriophages. One issue is the degradation of bacteriophages in the acid environment of the stomach, leading to limited oral bioavailability unless the administered bacteriophages are encapsulated [30] or the stomach acid is neutralized [19]. There is also evidence that bacteria that have established infection within the gut may be poorly accessible to bacteriophages, probably because they are located within the coating of the intestinal walls [31], leaving administered bacteriophages to simply pass by them on their way through.

**Issues with systemic delivery:** Although there is considerable evidence of efficacy in model systems [20,21], bacteriophages delivered via the circulation are challenged by a number of issues [32]. Both innate and adaptive immunity are major concerns. Bacteriophages are prime candidates for those elements of the innate response intended to provide a first response to invading viruses, notably phagocytosis [33]. In addition, the adaptive immune response will, in time, respond to bacteriophages, particularly after repeated administration. However, the ablative effect of such responses on bacteriophage efficacy appears to be limited [34]. Another major issue is delivery to the site of infection. Although bacteriophages have the unique ability to amplify locally even from a very small initial dose [11,23], they nevertheless have to reach and infect their target bacteria in order to be able to do so. Barriers that can be crossed by conventional antibiotics may be impervious to bacteriophages, favouring topical uses or use in body activities. However, a recent in-depth review of the subject noted bacteriophages as one of the most promising approaches to combating antimicrobial resistance even in systemic applications [7].

## 3. Bacteriophages

**Intellectual property issues:** It has long been argued that the patenting of phage therapy approaches is challenging. It is undeniable that the basic approach of phage therapy is firmly in the public domain. However, this is also true for monoclonal antibodies, and these have led to drugs with current market values in the tens of billions of dollars. It is also true that novel bacteriophages can be identified for almost any bacterial target, potentially bypassing patent protection. Again, this is also true for monoclonal antibodies. From a commercial point of view a patent is useful, but not actually essential since the progress of a drug or phage mix through the regulatory process underpins much of its value. However, patent protection is highly desirable, particularly in the early stages of development. There is considerable confusion over the patenting of naturally occurring biological materials [35], but it is clear that bacteriophage mixtures exhibiting activities different from those seen in nature can form the basis for patent awards.

**Bacteriophages are available:** It should go without saying that the bacteriophages used must be able to target the infecting bacteria, whether as part of a broadly effective cocktail [11] or by selection for the bacterial strain(s) present [36]. While it is thought that bacteriophages exist for all bacteria, they are sometimes hard to find, as with certain members of the *Streptococci* [37]. Alternatively, properties of the host bacteria can make isolation of bacteriophages difficult, as with slow-growing members of the *Mycobacteria*. However, for many bacterial species, isolation can be both rapid and simple.

**Bacteriophages are effective in killing the target bacteria:** While some bacteriophages cause rapid killing of their host, others do not. Selection of those which produce rapid lysis and liberate large numbers of progeny bacteriophage (high burst size) is usually assessed in vitro. The normal method for this initially is to monitor the formation of plaques, selecting those bacteriophages that form large, clear plaques [38]. Growth in liquid culture, often using plate-based optical density systems, is also used for this purpose.

**Bacteriophages must target the bacteria responsible for the infection:** Most bacteriophages kill only a subset of strains within a single bacterial species. In order to kill sufficient members of a representative panel of strains of that species (a diversity panel), the activity of individual bacteriophages is tested against that panel and a mixture selected which provides broad coverage. This may be a mixture of broadly effective bacteriophages along with those selected to cover specific strains from a diversity panel, for example those with concerning levels of resistance to conventional antibiotics. While 100% coverage of a large diversity panel is not usually required (for antibiotics or for bacteriophages), there are minimum levels (analogous to those defined for antibiotic development) which need to be attained for a generally applicable therapy. It is also advantageous to ensure that as many strains as possible are targeted by multiple bacteriophages, minimising the potential for resistance.

**Cross-resistance:** Generation of resistance in vitro may be used to confirm that cross-resistance to candidate therapeutic bacteriophages in a cocktail (implying similar biology of infection) is minimized. This involves the selection of bacteria which do not show similar resistance profiles against bacteriophage-resistant mutants of the target bacteria, and (as with antibiotics) minimizes the potential for the development of resistance in vivo.

**Coverage:** Isolates from different pathologies and geographic locations must be covered by the bacteriophages selected if a standardized cocktail is to be used [11,19,22,24,25,26], and this can be difficult to attain with some highly variable bacterial species. An alternative approach, of having a panel of bacteriophages with defined activities, from which personalized cocktails are prepared for individual patients [36,39] faces a number of currently unresolved regulatory challenges, as well as significant resourcing issues.

**Bacteriophages are obligately lytic, not temperate:** Obligately lytic bacteriophages that infect a permissive host produce rapid killing. The bacterial life cycle is simply “kill or be killed”. However, in many cases bacteriophages are temperate. That is, they are capable of entering a latent state within the host bacteria (lysogeny) in which they are usually integrated into the bacterial genome. In such a state, host bacteria are often rendered resistant to superinfection with the same, or even closely related, bacteriophages [40,41]. As and when a lysogenic bacteriophage reactivates, it may carry bacterial genes with it to a new host, a process known as specialized transduction. In addition, it is now becoming clear that lysogenic bacteriophages may modulate genetic activity and actually benefit their bacterial host [40]. Thus, while lysogenic bacteriophages may reduce bacterial numbers both in vitro and in vivo [42], they are generally considered unsuitable for therapeutic use. Traditional tests for lysogeny include the formation of turbid plaques, where the clearance zone is clouded by surviving bacteria. More recently, genetic analysis can identify markers of lysogeny such as the presence in the bacteriophage genome of a functional integrase gene or known repressors linked to the establishment and maintenance of a lysogenic infection state. This then allows exclusion from the development pathway. For some bacteria, such as *Clostridium difficle*, all bacteriophages identified to date appear to be temperate [43], limiting the options for phage therapy in these settings.

**Transduction and toxins:** As well as the specialized transduction seen in lysogenic infections, bacteriophages can pick up a wide range of segments of bacterial DNA essentially at random and transfer them to a new host. This is considered undesirable in candidate therapeutic bacteriophages since virulence genes could be transferred by this route. In addition, some bacteriophages actually carry such genes, including both antibiotic resistance genes [44] and clinically significant bacterial toxins [45], while others have adapted forms that transfer virulence-associated genes [46]. It is necessary to exclude such bacteriophages from therapeutic development, usually by genetic analysis.

**Stability:** The most promising therapeutic bacteriophage is of no value if it loses activity too fast before it can be used to treat patients. Assessment of stability in suitable forms for therapeutic use is, thus, a vital part of development [47].

**Quality of product:** In order to be used as therapeutics, bacteriophages must be able to grow to sufficiently high titres in a suitable bacterial host. Such hosts are often selected for their non-pathogenic nature, including absence of toxins and even of lysogenic bacteriophage genomes. It is however necessary to ensure that the bacteriophages produced by such compliant hosts retain their virulence against target bacteria. Unlike early therapeutic products, it is also necessary to purify bacteriophages away from host bacterial components, in particular the endotoxins produced by Gram-negative bacteria for which strict limits are applied [48,49]. The whole issue of producing bacteriophages to the required standards (as a drug substance) then combining them to make a therapeutic cocktail (i.e., a drug product) while complying with necessary quality control standards (cGMP, except for all but the earliest trials) is both demanding and expensive. However, if such standards cannot be attained, perhaps because of a persistent contaminant, then all of the preceding work has been in vain.

**Bacteriophages as biological control agents:** Bacteriophages are not chemical antibiotics. Although this statement seems obvious, it has not stopped some groups from assuming that the same processes used in the development of chemical antibiotics must of necessity be applied a priori to therapeutic bacteriophages. While bacteriophages do need to conform with existing rules, the regulators show a significant degree of flexibility, and indeed enthusiasm, in working with these new paradigms [50,51,52,53]. This is an area which is likely to see significant changes. However, these will rely on good science and, in particular, on data from fully regulated clinical trials, which are still sparse in this area. One important difference to conventional antibiotics is that bacteriophages are unlikely to produce sterilising effects in vivo since, when bacterial numbers fall below the replication threshold [29], they will lose much of their effect. However, in such situations, lowering bacterial numbers far enough can inhibit their replication [52] and reduce toxin production, thus allowing the immune system of an infected patient to aid in resolving the infection [11,22].

## 4. Stages of target selection

**Figure 2:**
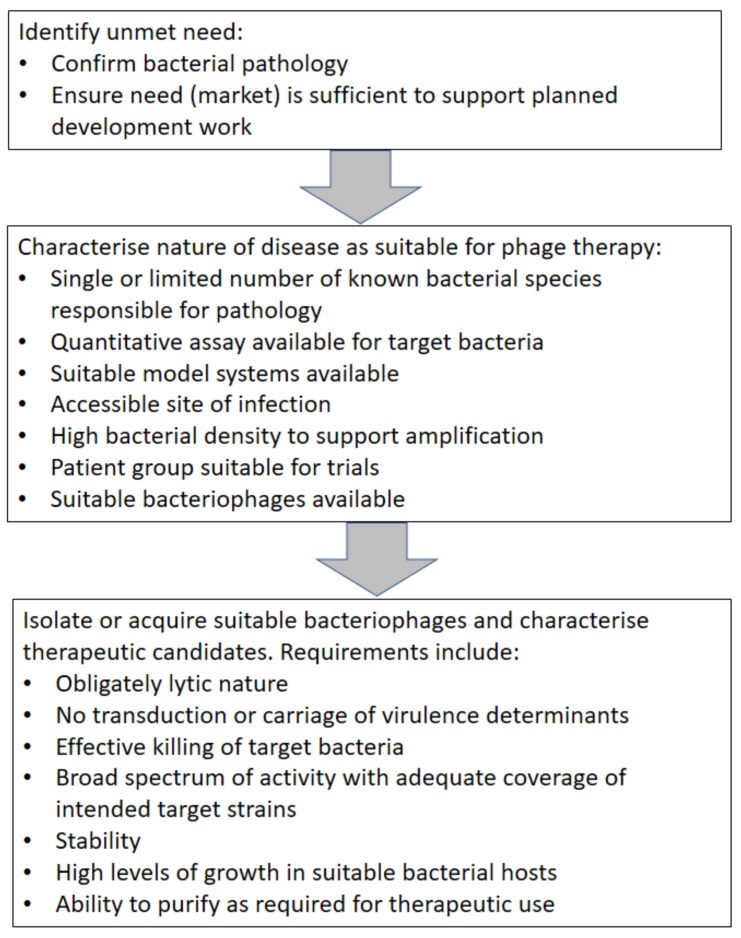
Steps in the selection of an initial target disease for phage therapy.

The following stages need to be considered (see Figure 2):

## 5. A Worked Example

As an example of the practical application of such considerations, *P. aeruginosa* appears to highly suitable as a phage therapy target, as outlined below and elsewhere [54]. As a result, it is proving a popular target for such intervention [11,22,23,24,25,26]. Indeed, the only successful phase 2 trial reported to date targeted late stage ear infections caused by *P. aeruginosa* [11].

In this study [11] the bacterial pathology had been confirmed both generally and in the specific cases by repeated microbiological assessment. The individual clinical need was severe (aiding patient recruitment and ethical review) and the market need was assessed as sufficient to support development, with the potential for expansion into other types of topical infection thereafter. While ear infections are initially polymicrobial, use of existing therapies that fail to clear the infection typically results in a “late-stage” infection dominated by *P. aeruginosa*, which was assessed prior to trial entry. A suitable trial centre was identified with strong clinical expertise, at which patients were accessible and willing to participate. *P. aeruginosa* is relatively simple to culture and to enumerate, and a previous veterinary field trial [22,55] had provided useful preclinical data. The ear is accessible, allowing direct application of bacteriophage onto the infected site, and had a high density of *P. aeruginosa* (2.3 × 10^6^ to 4.5 × 10^10^ CFU/gram at the start of the trial, averaging well over 10^9^ CFU/gram) [11]. Bacteriophages specific for *P. aeruginosa* are relatively widespread and simple to isolate from available sources [54]. Obligately lytic forms are common, showing rapid bacterial killing and releasing high numbers of progeny bacteriophages. Levels of transduction or toxin carriage are low, and broad activity of bacteriophages against *P. aeruginosa* is obtainable. The bacteriophages could be grown in culture to the required level and purified from such cultures. Though the presence of endotoxins in lysate of Gram-negative bacteria such as *P. aeruginosa* does require careful consideration of levels remaining in a candidate therapy, the low dosing levels used in the study (a single input dose of 2.4 ng) aided in this. With due consideration given to the above elements, the phase 1/2 clinical trial was conducted (albeit at a small scale) [11] and, uniquely to date, produced promising and positive results.

## 6. Summary

Phage therapy remains experimental in both human and veterinary medicine. With antibiotic resistance now acknowledged as a worldwide crisis [4], the need for new approaches to antibacterial therapy makes the development of this powerful approach a priority. Despite this, there is a real shortage of high quality clinical work on which to base progression of phage therapy through the regulatory approvals required to permit widespread use.

The selection of appropriate disease targets is an essential step in progressing this important technology. Trial failures are expensive in both time and resources and benefit nobody. Such failures carry the risk of causing serious damage to confidence in the field as a whole [15]. In order to maximise the chances of success, the selection of disease targets must be informed by sound knowledge of the disease to be treated, of the infecting bacteria, and of the nature and interactions of the bacteriophages to be used. Success against carefully selected initial targets is required to build the confidence to allow later work in more challenging applications

Based on the work to date, while it seems likely that bacteriophages will be used systemically and even orally in time, topical treatments where the bacteriophage can be placed onto an infection site with a high bacterial density offer the best chance of success. Such success is vital to moving phage therapy into clinical use, to save lives, and to improve lives.
